# Smartphone Motion Sensor-Based Complex Human Activity Identification Using Deep Stacked Autoencoder Algorithm for Enhanced Smart Healthcare System

**DOI:** 10.3390/s20216300

**Published:** 2020-11-05

**Authors:** Uzoma Rita Alo, Henry Friday Nweke, Ying Wah Teh, Ghulam Murtaza

**Affiliations:** 1 Computer Science Department, Alex Ekwueme Federal University, Ndufu-Alike, Ikwo, P.M.B 1010, Abakaliki, Ebonyi State 480263, Nigeria; alo.uzoma@funai.edu.ng; 2 Computer Science Department, Ebonyi State University, P.M.B 053, Abakaliki, Ebonyi State 480211, Nigeria; 3 Department of Information Systems, Faculty of Computer Science and Information Technology, University of Malaya, Kuala Lumpur 50603, Malaysia; gmurtaza@iba-suk.edu.pk; 4 Department of Computer Science, Sukkur IBA University, Sukkur 65200, Pakistan

**Keywords:** activity identification, deep learning, acceleration sensor, smart healthcare, deep stacked autoencoder, health monitoring

## Abstract

Human motion analysis using a smartphone-embedded accelerometer sensor provided important context for the identification of static, dynamic, and complex sequence of activities. Research in smartphone-based motion analysis are implemented for tasks, such as health status monitoring, fall detection and prevention, energy expenditure estimation, and emotion detection. However, current methods, in this regard, assume that the device is tightly attached to a pre-determined position and orientation, which might cause performance degradation in accelerometer data due to changing orientation. Therefore, it is challenging to accurately and automatically identify activity details as a result of the complexity and orientation inconsistencies of the smartphone. Furthermore, the current activity identification methods utilize conventional machine learning algorithms that are application dependent. Moreover, it is difficult to model the hierarchical and temporal dynamic nature of the current, complex, activity identification process. This paper aims to propose a deep stacked autoencoder algorithm, and orientation invariant features, for complex human activity identification. The proposed approach is made up of various stages. First, we computed the magnitude norm vector and rotation feature (pitch and roll angles) to augment the three-axis dimensions (3-D) of the accelerometer sensor. Second, we propose a deep stacked autoencoder based deep learning algorithm to automatically extract compact feature representation from the motion sensor data. The results show that the proposed integration of the deep learning algorithm, and orientation invariant features, can accurately recognize complex activity details using only smartphone accelerometer data. The proposed deep stacked autoencoder method achieved 97.13% identification accuracy compared to the conventional machine learning methods and the deep belief network algorithm. The results suggest the impact of the proposed method to improve a smartphone-based complex human activity identification framework.

## 1. Introduction

Recent research in human activity recognition and ambient assisted living has revealed a high correlation between the level of physical activity and maintaining a healthy lifestyle [1]. Engaging in physical activity is vital toward reducing several chronic and non-communicable diseases, such as cardiovascular diseases, stroke, diabetes, hypertension, obesity, and depression. According to the World Health Organization (WHO), physical activity involves body movement produced by skeletal muscles that require energy expenditure. These include activities undertaken while working, playing, conducting household chores, and engaging in recreational activities [2]. The impact of physical activity for the overall well-being of an individual cannot be overemphasized. Moreover, regular physical activity can bring about many physiological improvements on an individual’s emotional state, help the aging population live active lifestyles, and enhance self-esteem. In addition, engaging in physical activity is important toward lowering stress and anxiety levels, reducing blood pressure, contributing toward weight loss, and reduce the risk of cognitive conditions, such as Alzheimer’s, in elderly citizens [3]. Certainly, lack of physical activity is the major cause of obesity, a condition that is currently ravaging the world, with over 10% of the world population being overweight. Therefore, precautionary measures, such as physical activity and appropriate dieting, are needed to reduce the impact of the current obesity level.

To assess the level of physical activity, nutritionists and medical practitioners implement self-completed questionnaire techniques to enable people to log their daily activities, and then analyze these questionnaires to make informed decisions, and recommend appropriate feedback. Nonetheless, the use of a self-completed questionnaire technique is laborious and time-consuming to evaluate, especially for large groups. Therefore, sensor based technologies have recently been adopted by researchers to identify levels of physical activity engagement [1,4,5]. Sensor-based methods for physical activity assessment offer superior processes for gathering and collecting everyday contextual information and life logging data [1,6].

In this case, the use of lifelogging data collected through sensor technologies provides a means to continuously capture individual experiences with varieties of sensor modalities, which can be stored in personal multimedia archives [6]. These sensor streams are analyzed to provide adequate information for physical activity identification and assessment, health monitoring, and other social interactions. Furthermore, advances in Internet of Things (IoT) devices with embedded sensors can also be used for complex physiological data collection through life logging, and intelligently analyzed for human activity detection, energy expenditure estimation, stress detection, and chronic disease management. Analysis of human contextual data further provide opportunities to develop effective and efficient smart healthcare systems.

The analysis of lifelogging data collected through sensor embedded devices for human activity identification and assessment can be traditionally implemented using video, wearable and ambient sensors, and smartphone-based approaches [7,8,9,10]. However, the performance of video-based methods are affected by lightening variabilities and sensor locations. Furthermore, issues, such as privacy and inability to differentiate target from non-target information during sensing, have drastically minimized successful implementation of the video-based method [11]. On one hand, wearable and ambient sensor-based methods provide continuous and consistent monitoring of physiological signals. However, the approach requires numerous sensors to be worn by subjects at different body positions, and might be unable to provide real-life practical implementation of daily activity details.

Recently, a smartphone-based method for human activity identification was shown to provide individuals with a better way to seamlessly track daily physical activities, in order to access energy expenditure, and maintain a healthy lifestyle. Smartphones are equipped with various sensor modalities, such as an accelerometer, gyroscope, magnetometer, Global Positioning System (GPS) compass, and other physiological signals. These sensor modalities provide unobtrusive, continuous, and real-time based human activity identification, and assessment. An activity identification framework using a smartphone can be categorized into simple or complex activity details. Simple activities are activities of daily living that are performed in a short period of time. Activities, such as walking, running, sitting, standing, and jogging, are classified as simple activities. Conversely, complex activities are composed of a sequence of activities performed at a longer duration of time and include smoking, eating, taking medication, cooking, writing, etc. [7,12].

Among the sensor modalities for human activity identification, the accelerometer-based sensor is the most widely implemented [13,14,15]. An accelerometer sensor utilizes the acceleration forces of the body to dynamically measure body movements and vibrations, which ensure automatic identification of movement patterns. The sensor has low power consumption that enables continuous monitoring of human behavior, and does not depend on external signal sources for effective human activity identification. In addition, an accelerometer sensor contains highly detailed information about phone movement and orientation [16,17]. However, the fundamental challenges faced by accelerometer-based human activity recognition include orientation variation and position displacement [18,19]. In this case, smartphones for data collection are assumed to be tightly attached or placed at a pre-determined location and orientation, and do not change during the activity identification process. Conversely, this is not the case in real-world activity identification using smartphones [19,20], as users are obligated to carefully place, or carry, the smartphone at a particular location. Therefore, the central theme of this study is to investigate approaches to solve orientation invariant problems in accelerometer-based human activity identification for smartphone users. We propose automatic feature representation using the deep learning model, and feature augmentation methods, to resolve the above issue.

Previous studies have investigated various methods to minimize the impact of orientation variation in mobile phone-based human activity identification. These methods include the use of rotation matrix to transform the signal into global reference coordinate systems, extraction of robust features that are independent of orientation, integration of an accelerometer with a gyroscope to reduce the impact of orientation, and user adaptation [19,21,22]. However, the rotational matrix transformation method has high computational complexity due to the extensive computation required to transform the sensors to a global reference coordinate system. On the other hand, small-scale errors in the output of a gyroscope sensor may lead to a drastic change in the sensor values for long time applications in the case of the sensor integration method. Furthermore, a magnetic sensor is power hungry and noisy, which might result in an inaccurate measurement reading [21]. In addition, it is difficult to correct the effects of the sensor drift that is prevalent in the gyroscope inertial signal. Early correction of the sensor drift is important for assessing the pre-impact fall detection and related application.

In many studies on orientation invariant problems, the orientation independent feature approach is widely utilized. In this case, the raw signal (three axis) is converted into one dimensional (1-D) orientation invariant sensors by computing the combined magnitude of the sensor axes. Then, orientation invariant features are extracted and fed to conventional machine learning models, such as the support vector machine, k-nearest neighbors, decision tree, and artificial neural networks for activity classification [7,18,23]. Nevertheless, traditional machine learning approaches are application dependent, and challenging to model complex activity details. Furthermore, they have shallow architecture, which might not be suitable for continuous activity identification. In addition, it is challenging to handle real-time activity identification and noise in large sensor data through conventional machine learning [24,25]. To resolve the above challenging issues in smartphone-based human activity identification, it requires robust, automatic feature representation methods.

Hence, deep learning methods were recently proposed by various studies to minimize the issues inherent in conventional feature learning methods [25,26,27]. Deep learning methods automatically extract translational invariants and discriminative features from large smartphone sensor streams, and have provided unprecedented performances in areas, such as image segmentation and processing, speech recognition, medicine, natural language processing, and human activity identification [26,28].

We proposes to augment the tri-axis (*X*, *Y*, and *Z*) acceleration data, with magnitude vector and rotation angle (pitch and roll) of the acceleration sensors. These methods are essential to provide orientation invariant and position independent features, to minimize orientation inconsistencies and effects of displacements. The unified method is efficient and robust against orientation changes in the smartphones with less computation time [29,30]. Specifically, the paper proposes the deep stacked autoencoder based deep learning method for automatic feature representation and orientation augmentation through magnitude vector and rotation angle for human activity identification. The deep stacked autoencoder is straightforward to create and implement, with great representational feature learning from smartphone sensor data. To the best of our knowledge, no other studies have evaluated the impact of magnitude vector, pitch and roll values, for deep learning-based human activity identification.

Accordingly, the contributions of this paper are as follows:

- Propose a deep stacked autoencoder based deep learning algorithm for complex human activity identification using smartphone accelerometer data to improve accuracy and reduce over-fitting.
- Investigate the impact of fusing magnitude vector and rotation angle (pitch and roll) with tri-axis (3-D) accelerometer data to correct the effects of smartphone orientation on complex human activity identification.
- Extensive experimental settings to evaluate the proposed method using a smartphone acceleration sensor placed on the wrist, and pocket, and compare the performance with three conventional machine learning algorithms (Naïve Bayes, support vector machine, and linear discriminant analysis), and deep belief networks.

The rest of the paper is organized as follows. Section 2 presents the related works on deep learning for human activity identification. Section 3 discusses the methodology, which includes pre-processing, deep stacked autoencoder, and baseline conventional machine learning methods. We present the experimental design, dataset description, and evaluation metrics in Section 4. Section 5 discusses the result of the findings and compares them with the existing deep learning model. We draw conclusions and outline directions for future research in Section 6.

## 2. Related Works

This section presents relevant literature on human activity recognition using mobile and wearable sensor data. The section provides a brief overview of studies that utilized conventional machine learning and deep learning methods for human activity recognition, as well as strengths and weaknesses of the studies.

### 2.1. Conventional Machine Learning Methods for Human Activity Identification

In past decades, various studies have proposed conventional machine learning and feature extraction methods for human activity detection. Initially, studies extracted feature vectors, such as time and frequency domain features from smartphone sensors after data preparation, and fed to classification algorithms for detection of activity details. Typical activity classification algorithms that have played prominent roles in this regard include the support vector machine [6,11,29], Naïve Bayes [7,11], logistic regression [11], K nearest neighbors [7,11,31,32,33], decision tree [1,7], clustering method [34,35], linear discriminant analysis [36], etc. These approaches provide an effective mechanism to implement real-time recognition of activity details. However, conventional approaches require extensive feature extraction and selection methods that are often arbitrary, and lack generalization ability to model the hierarchical and temporal dynamic nature of the current human activity identification process. In addition, conventional feature methods provide no universally acceptable feature vectors that accurately represent datasets and applications [26]. Moreover, it is challenging to model real-time activity identification and noise in large sensor data [24]. To solve the problem, inherent in current traditional machine learning methods, requires sophisticated and automated feature representation using deep learning methods.

### 2.2. Deep Learning-Based Human Activity Recognition

Recently, deep learning methods have gained popularity for their ability to automatically extract translational invariant features from sensor data, in order to resolve some of the issues in conventional feature learning methods. Deep learning methods use artificial intelligence techniques and multiple layers of neural networks to automatically extract features from large smartphone sensor streams. In addition, deep learning methods exploit the massive sensor streams generated through various cyber-physical systems, Internet of Things (IoT), and powerful hardware resources, such as graphical processing units (GPU) to build human activity identification frameworks. The main advantages of deep learning methods are their ability to deploy multiple layers of neural networks (deep neural networks) and unlabeled sensor streams to achieve higher accuracy, fast processing, and translational invariant feature representation for human activity identification.

Recently, various studies have attempted to implement deep learning for automatic feature representation and human activity identification using smartphones [26]. Hassan, et al. [37] proposed deep belief network and principal component analysis (PCA) to model human activity recognition through features extracted from smartphone sensors. In a related approach, Alsheikh, et al. [38] proposed deep belief networks to extract hierarchical features from motion sensor data and then modeled stochastic temporal activity sequence using the Hidden Markov model. In an attempt to build real-time and automatic feature representation for human activity identification, Bhattacharya and Lane [39] investigated smartwatch and smartphone-based activity detection using GPU enabled devices. The proposed method was deployed for hand gesture recognition, indoor localization, and transport mode detection. Varieties of other deep learning algorithms, such as convolutional neural networks (CNN) and Recurrent Neural Network (RNN) have also been implemented for smartphone human activity. For instance, recent works in [14,40,41] explored the performance of convolutional neural networks for smartphone-based human activity identification and indoor localization. In their studies, CNN methods were deployed to automatically extract translational invariant and temporal features from accelerometer sensors collected with smartphones, while the subjects executed various simple activities. Moreover, the CNN method has shown impressive results in identification of simple activities using accelerometer and gyroscope sensors [42]. Similarly, Almaslukh, et al. [8] investigated online smartphone-based implementation of deep convolutional neural networks for real-time human activity identification. Specifically, utilizing data augmentation and local connectivity of CNN, the proposed method outperformed conventional approaches using principal component analysis, random forest, and support vector machine algorithms.

Recurrent neural networks based deep learning algorithms have also been evaluated for smartphone-based human activity recognition. Recurrent neural network methods ensure extraction of long range temporal features from raw motion sensors in order to automatically model activity sequences. Various studies have explored implementation of recurrent neural networks, such as long-short memory, gated recurrent units, and bi-direction long short-term memory for human activity identification and related applications [43,44]. Zhao and Hou [9] explored the bidirectional long short-term memory (Bi-LSTM) approach for real-time human activity identification using a smartphone sensor, and investigated the possibility of onboard implementation for android devices. These studies have shown the potential of deep learning algorithms to provide efficient and effective approaches to implement human activity identification using smartphone motion sensors.

However, a number of issues have hindered successful implementation of the aforementioned deep learning algorithms for smartphone-based applications. For instance, deep learning algorithms using deep belief networks require high parameter initializations that may be computationally expensive for mobile-based activity detection frameworks. In addition, joint optimization to achieve high generalization is practically difficult. Even though convolutional and recurrent neural network based methods are widely implemented, especially for image and video-based applications, it requires high parameter initialization to achieve optimal results, and may over-fit easily when using complicated fully connected layers [45]. In addition, it is challenging to capture temporal variance and change in activity details due to lack of temporal segmentation in convolutional neural networks. To model temporal dependencies in activity details requires fusion with recurrent neural networks, which further increases the computational complexity of activity identification models [26]. Moreover, a recurrent neural network is difficult to train and suffers from vanishing gradients.

Recently, a few studies [46,47,48,49,50] have implemented a deep stacked autoencoder for human activity identification. Nonetheless, these studies assume that the smartphone is tightly attached or placed at a pre-determined position, and that orientation does not change during the activity identification process. Conversely, this is not the case in real-world activity identification using smartphone sensors, especially with accelerometer sensors [19,20], as users are obligated to carefully place or carry the smartphone in particular positions. Various studies in orientation invariance and sensor displacement have been implemented recently [7,18,30]. Conversely, these studies utilized the conventional feature extraction and machine learning approach.

This paper differs distinctly from previous studies by proposing a unified deep learning method and orientation invariant features for complex human activity identification. Complex human activity details are representative of peoples’ daily lives, and are harder to recognize. Identification of complex activity details is necessary for building a real-life smartphone-based human activity identification application. To the best of our knowledge, this is the first work revealing the impact of the deep learning approach and orientation invariant features for a smartphone-based complex human activity identification framework. Moreover, this paper implements extensive experiments to compare the performances of tri-axis acceleration, magnitude vector, and rotation angle (pitch and roll) separately, and fused together. The experimental evaluations suggest the impact of the proposed deep learning approach for human activity identification. Table 1 summarizes various deep learning models recently implemented for human activity recognition, strengths, and weaknesses.

## 3. Methodology

**Figure 1:**
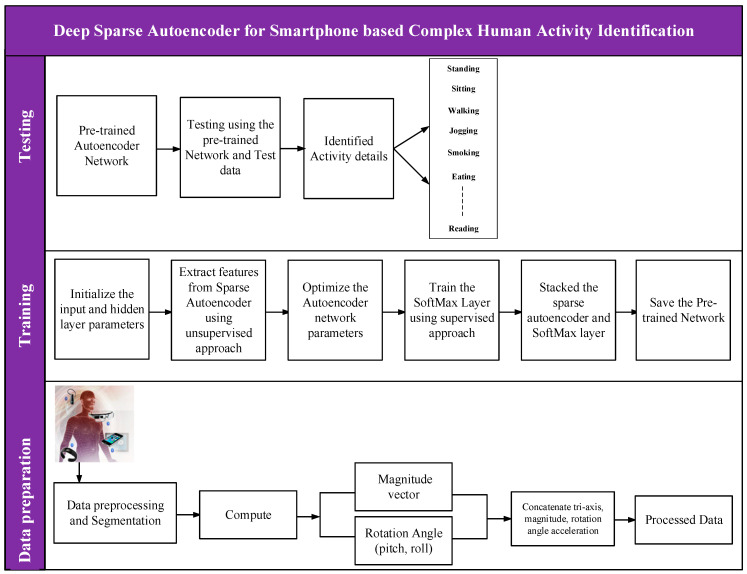
Proposed complex human activity identification framework.

This section describes the methodology adopted to develop the deep learning-based complex human activity identification. The section is divided into various subsections, and includes the pre-processing method and theoretic concept of the proposed deep stacked autoencoder and orientation invariance methods for human activity recognition. In addition, we briefly explain the methods recently implemented for human activity recognition that we compared with the proposed method. The details flow of the proposed approach is presented in Figure 1.

### 3.1. Pre-Processing

We use two main techniques for data pre-processing, data cleaning, and data segmentation. Raw signal sensor data collected using smartphones and other wearable devices are corrupted by noise and missing values, due to signal degradation or signal variation generated during the sequence of activities performed by each subject. Therefore, noise removal and filtering are required before feature extraction and activity identification algorithms. Filtering methods are essential to remove low-frequency data, geometric bias of the sensor dimension, and to enhance the correlation between each data point. We applied linear interpolation [29] to the raw accelerometer data to input missing values and replace the values at the end of the activity sequence with previous values in the activity data.

To ensure efficient smartphone and wearable device implementation, data for activity identification are divided into a series of data points, termed segments. Here, we developed and applied the sliding window without overlap approach to the processed data. The sliding window method is efficient and has become the preferred data segmentation method for human activity identification [52]. However, choosing the size of the sliding window is a contentious issue in human activity identification and classification. The window sizes have a great impact on computation time and identification of activity details. Ambulatory and repetitive activities, such as walking, jogging, running, etc., require lower window sizes, while other activity details, such as typing and smoking, may be recognized with longer window sizes. In a recent study, Shoaib et al. [7] evaluated different window sizes, such as 2, 5, 10, 15, 20, and 30 s, and concluded that window sizes in the ranges of 2–5 s are sufficient for complex activity identification. We empirically set the window size and implement the segmentation procedure using previously tested window sizes for human activity recognition. Therefore, we used a 2 s window size (100 sample with 50 Hz sampling rate) without overlapping between each sample, as recently utilized in [7].

### 3.2. Deep Learning Framework for Complex Human Activity Identification

In this paper, the autoencoder algorithm is proposed for complex human activity identification using acceleration sensor data. The deep stacked autoencoder method is straightforward to create and implement. Moreover, the deep stacked autoencoder method uses the unsupervised feature learning approach and describes expressive abstraction with great representational feature learning from mobile sensor data. The theoretical framework behind the proposed deep stacked autoencoder is presented below.

#### 3.2.1. Autoencoder

Deep autoencoder-based deep learning is a generative feature learning method that replicates the copies of the input values as outputs. Therefore, the method is efficient for dimensionality reduction of complex and high-dimensional sensor streams generated using mobile and wearable devices for human activity recognition. The deep autoencoder is divided into the input layer, encoding layer, decoding layer, and output layer. The encoding layer transforms the sensor streams generated by the smartphone device into hidden features. Then, the transform input features is reconstructed by the decoding layer to approximate values to minimize reconstruction errors [26]. With these approaches, the deep autoencoder provides a data-driven mechanism for learning feature extraction to minimize over-dependence on handcrafted features inherent in conventional machine learning methods. Given the input matrix from the acceleration sensor $X_{n}=\{x_{1},x_{2},\ldots \ldots ..x_{n}\},x_{n}\in R^{D}$ and the corresponding activity class label $Y_{n}=\{y_{1},y_{2},\ldots \ldots ..y_{n}\},y_{n}\in R^{D}$. The encoding layer linearly maps input data to the hidden layer $h_{k}$ using the logistic sigmoid activation function σ. The encoding process is represented as

(1) $$h_{k}=\sigma (w_{k}{}^{\left(e\right)}.x_{k}+b_{k}{}^{\left(e\right)})$$

where $(w_{k}{}^{\left(e\right)},b_{k}{}^{\left(e\right)})\in R^{D}$ represents the weight matrix and bias vector, $h=\{h_{1},h_{2},h_{3},\ldots ..h_{k}\}$ represents the output of the hidden layers, and *x* is the sensor input values. Furthermore, the decoding layer aims to reconstruct the input in the output layers. The reconstruction process is represented as

(2) $$y_{k}=\sigma (w_{k}^{\left(d\right)}.h_{k}+b_{k}^{\left(d\right)})$$

$(w_{k}^{\left(d\right)},b_{k}^{\left(d\right)})\in R^{D}$ represents the weight matrix and bias vector of the reconstruction process. Next, the autoencoder tries to minimize the error between the input matrix $X_{n}$ and $Y_{n}$ by applying the objective loss function. The objective loss function is represented as:

(3) $$J(w_{k}^{\left(e\right)},b_{k}^{\left(e\right)},w_{k}^{\left(d\right)},b_{k}^{\left(d\right)})=\frac{1}{2}{\left\|Y_{n}-X_{n}\right\|}^{2}$$

This can further be expressed as

(4) $$Y_{n}=h(X_{n}/w_{k}^{\left(e\right)},b_{k}^{\left(e\right)},w_{k}^{\left(d\right)},b_{k}^{\left(d\right)})$$

rewriting with respect to Equation (6), gives

(5) $$J(w_{k}^{\left(e\right)},b_{k}^{\left(e\right)},w_{k}^{\left(d\right)},b_{k}^{\left(d\right)})=\frac{1}{2}{\left\|h(X/w_{k}^{\left(e\right)},b_{k}^{\left(e\right)},w_{k}^{\left(d\right)},b_{k}^{\left(d\right)})-X_{n}\right\|}^{2}$$

In our study, the stacked autoencoder was used to develop the deep learning-based complex human activity identification framework using smartphone acceleration sensor data. The concept of sparsity in autoencoder-based deep learning is explained in the next subsection.

#### 3.2.2. Deep Sparse Autoencoder

Sparse autoencoder [53] is an unsupervised deep learning method that learns over-complete feature representation from raw sensor data by utilizing the sparsity term to model loss function, and set some of the active units to zero. The use of sparsity term allows the model to learn feature representations that are robust, linearly separable, and invariant to changes, distortion, and displacement, and learning applications. These significant characteristics of sparse autoencoder guarantee efficient extraction of low dimensional features from high-dimensional input sensor data. In addition, it ensures compact representation of a complex activity recognition framework. In this paper, we implemented a three-layer sparse autoencoder trained in greedy-wise layer approach. Thus, the output of the first layer serves as input to the second layer. The weight matrix, bias vector, and loss function of the model are iteratively updated at each training iteration. The sparsity term is added to the autoencoder cost function using the regularization term. The use of greedy-wise training helps to determine the average output activation value of the network in order to minimize overfitting [26]. The regularization term on the weight and sparsity constraint added to the network is shown in Equations (6)–(9) below:

(6) $$J(W_{k}^{\left(e\right)},b_{k}^{\left(e\right)},W_{k}^{\left(d\right)},b_{k}^{\left(d\right)})=\frac{1}{2}\left\|h(X_{n}|C_{1}\right\|+\varphi C_{2}+\gamma C_{3}$$

(7) $$C_{1}=W_{k}^{\left(e\right)},b_{k}^{\left(e\right)},W_{k}^{\left(d\right)},b_{k}^{\left(d\right)}-X_{n}$$

(8) $$C_{2}=\varphi \sum_{j}KL(\rho ,{\rho^{\prime }}_{j})$$

(9) $$C_{3}={\left\|W_{k}^{\left(e\right)}\;W_{k}^{\left(e\right)}\right\|}_{2}^{2}$$

where φ and γ represent $L_{2}$ regularization coefficient for the weight matrix, sparsity regularization factors that control the degree of weight decay, and KL() is the Kullback–Leibler divergence, respectively. The sparsity regularization is added to the network to restrict the sparsity control of the hidden units [53]. Then, Kullback–Leibler divergence, which measures the diversity of two training sample distribution, is computed as

(10) $$KL(t,m)=t.\log(\frac{t}{m})+(1-t).\log(\frac{1-t}{1-m})$$

ρ is the probability of activation and ${\rho^{\prime }}_{j}$ is the average activation probability of j−th hidden neuron [54].

#### 3.2.3. Softmax Classifier

At the end of feature extraction using a deep sparse autoencoder, the Softmax classifier is appended at the fully connected layer to provide probability distributions of activity classes. The Softmax classifier is a multinomial logistic regression that models multi-class classification problems using the cost minimization method. Hence, given the sensor data and the corresponding activity class label $\left(x_{n},y_{n}\right)$, where $x_{n}\in \left[1,2,3\ldots \ldots .k\right]$ and $y_{n}\in \left[1,2,3\ldots \ldots .p\right]$. In our dataset, p is total of thirteen (13) activity classes, which include walk, stand, jog, sit, bike, upstairs, downstairs, type, write, coffee, talk, smoke, and eat. Given the input matrix $x_{n}$ and $y_{n}$, the Softmax model estimates the probability p(y=p/x) for each class label p=1,2,3,….p for p class problem. The probability estimate contains each activity class probability that measures the input values for each activity class. The probability estimate of each class $h(x_{i}/\theta )$ is computed as

(11) $$h(x_{i}/\theta )=\left[\begin{array}{c} p(y=walk/x_{i};\theta )\\ p(y=s\tan d/x_{i};\theta )\\ p(y=sit/x_{i};\theta )\\ :\\ p(y=eat/x_{i};\theta ) \end{array}\right]$$

(12) $$=\frac{1}{\sum_{j}^{p}\exp(\theta_{j}^{T}x_{i})}\left[\begin{array}{c} \exp(\theta^{(walk)T}x_{i})\\ \exp(\theta^{(s\tan d)T}x_{i})\\ \exp(\theta^{(sit)T}x_{i})\\ :\\ \exp(\theta^{(eat)T}x_{i}) \end{array}\right]$$

where $\theta^{\left(walk\right)},\theta^{\left(s\tan d\right)},\theta^{\left(sit\right)},\ldots ..,\theta^{\left(eat\right)}\in R^{n}$ depict the parameters of the stacked autoencoder model for each activity class. The normal distribution [55] is given as:

(13) $$\frac{1}{\sum_{j}^{p}\exp(\theta_{j}^{T}x_{i})}$$

The Softmax classifier tries to optimize the θ parameters by minimizing the cost function, which is given as

(14) $$J(\theta )=\frac{1}{m}\left[\sum_{i=1}^{m}\sum_{j=1}^{p}1\{y(y-j)\}\log\frac{\exp(\theta_{j}^{T}x_{i})}{\sum_{i}^{p}\exp(\theta_{i}^{T}x_{i})}\right]+Q$$

(15) $$Q=\frac{\lambda }{2}\sum_{i=1}^{p}\sum_{j=0}^{n}\theta_{jp}^{2}(\lambda >0)$$

**Figure 2:**
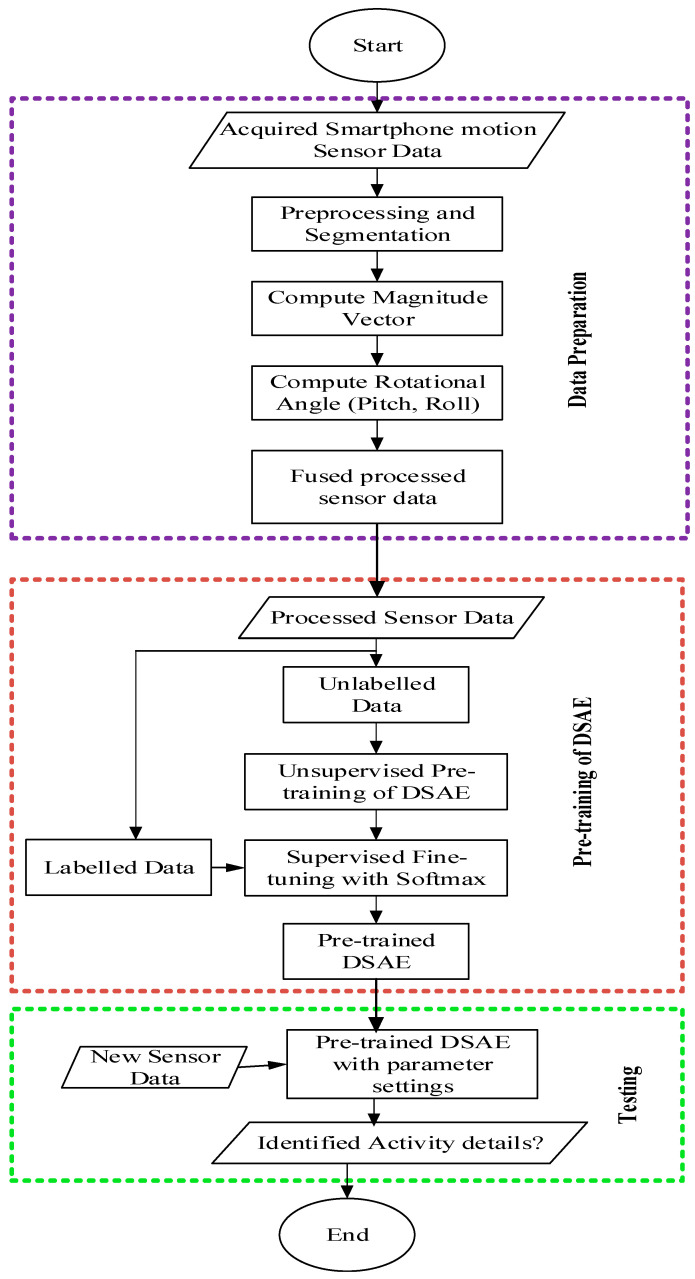
Flowchart of the proposed method.

Then, we fine-tuned the deep sparse autoencoder model with supervised methods to minimize the likelihood function and improve adaptability [56]. Finally, we stacked the pre-trained deep stacked autoencoder embedded with Softmax classifier layers, alongside the model weights, and all other model parameters. The weights and network parameters were updated concurrently in each iteration using the scaled conjugate gradient descent [57]. Algorithm 1 shows the training procedure of the proposed deep stacked autoencoder for complex human activity identification. Figure 2 depicts the overview of the proposed methods.

**Algorithm 1.** Deep learning-based complex activity identification training procedure. 1: Input: Training acceleration sample $X_{n}$ 2: Output: Set of activity detail performances 3: Sensor Data preparation 4: Obtain the acceleration sensor data from smartphone 5: Segment the sensor data using sliding window 6: Compute the magnitude using Equation (16) 7: Compute the pitch-roll values using Equations (17) and (18) 8: Network Parameter Settings 9: Set the number of hidden layers and neurons 10: Max epoch values 11: Sparsity regularization values 12: Train the stacked autoencoder using greedy-wise layer approach 13: Compute the cost function of the autoencoder algorithm at each layer using Equations (3)–(5) 14: Set the sparsity regularization values using Equations (6)–(9) 15: Obtain the network output 16: Stack the pre-trained network with their parameter values 17: Train the Softmax classifier to estimate their parameters 18: Minimize the cost function 19: Fine-tune the stacked autoencoder network weights using gradient descent 20: Obtain the activity details

### 3.3. Orientation Invariance in Smartphone-Based Human Activity Recognition

One of the major challenges in smartphone-based human activity identification is how to solve sensor orientation and sensor displacement related issues. Sensor displacement involves the change in sensor position, with respect to initial placement position, while sensor orientation automatically changes data distribution and drift. Variation in sensor orientation affects activity pattern, thereby, making it difficult for previously trained models to identify target activities in the observed sensor data [18]. The impacts of sensor orientation and displacement are highly noticeable in accelerometer-based human activity recognition [20]. Therefore, recent works have focused on methods to achieve robustness to sensor orientations. Methods proposed in literature include the use of orientation independent features, raw input sensor transformation from coordinate systems of the mobile phone into global coordinates prior to feature extraction, and unsupervised adaptation [19]. Moreover, robustness to sensor orientation has been resolved through fusion of the accelerometer and gyroscope signal, to correct the effect of orientation inconsistencies [18,21]. For instance, Ustev [58] proposed the fusion of acceleration, compass, gyroscope, and magnetometer to resolve the issue of orientation inconsistency. However, small-scale errors in the output of the gyroscope sensor may lead to a drastic change in the sensor values for long-time applications. Furthermore, the magnetic sensor is power hungry, and noisy, which may result in an inaccurate measurement reading [21]. In addition, it is difficult to correct the effects of the sensor drift that is prevalent in the gyroscope inertial signal. Early correction of the sensor drift is important for assessing pre-impact fall detection and related application.

To provide robust and orientation invariant representation for a complex activity identification framework, we investigate the use of three-axis sensor augmentation using the $L^{2}$ norm (magnitude) vector. Here, the raw signal is converted into one dimensional (1-D) orientation invariant sensors by computing the combined magnitude (MAG) of the sensor axes. Secondly, we adopt the position independent method to augment the accelerometer data by computing the pitch-roll angle of the acceleration signal [30]. The orientation of the smartphone at different placement positions may be subject to rotational variation, which automatically changes the information obtained at different positions. Consequently, computing the pitch-roll angle of the accelerometer will help to reduce the effect of rotation.

For a given signal generated by the smartphone, and watch, placed on the subject’s pocket, or wrist, during data collection, it generates a three-axis accelerometer signal when a particular activity is being performed. The signal generated by the subject can be represented as $s_{k}={\left(s_{x},s_{y},s_{z}\right)}_{k}^{T}$, where *x*, *y*, and *z* represent the axis of the acceleration signal, *T* is the time frame and *k* is the individual signal point. On the contrary, for another acceleration signal placed on the different position of the subjects, the relative sensor orientation between the two signals can be described by the rotation matrix $R^{\prime }$ and generate different signal ${s^{\prime }}_{k}$ where ${s^{\prime }}_{k}=R^{\prime }s_{k}$. The rotation of the signal sequence is expressed as follows $S_{r}=\langle s_{k}\rangle (k=1,\ldots \ldots ..,M_{s})$. When the matrix $R^{\prime }$ is rotated, it would result in different signal sequences, represented as ${S^{\prime }}_{r}=\langle {s^{\prime }}_{k}\rangle (k=1,\ldots \ldots ..,M_{s})$, where $M_{s}$ is the number of data points in the sequence, and r depicts the pocket or wrist acceleration signal. The transformation is denoted as $R^{\prime }⊙S_{r}:=\langle R^{\prime }s_{k}\rangle =s_{r}$ [21].

Therefore, using only the three-axis (*x*, *y*, and *z*) of the accelerometer data would result in orientation inconsistency. The change in data distribution as a result in orientation inconsistency would greatly impact the performance of the complex activity identification framework. We computed the $L^{2}$ norm (magnitude vector) of the accelerometer data using Equation (16):

(16) $$M_{i}=\sqrt{(s_{x}^{2}+s_{y}^{2}+s_{z}^{2}}{)}_{i}$$

In the same way, to counter the impact rotational changes, additional feature vector dimensions of the acceleration signal was computed using the pitch-roll angle.

(17) $$Pitch=\frac{180}{\pi }.a\tan2(s_{y}/g,s_{z}/g)$$

(18) $$roll=\frac{180}{\pi }.a\tan2(s_{x}/g,s_{z}/g)$$

where $s_{x}$$s_{y}$$s_{z}$ represent the individual axis of the accelerometer data, g is the gravitational acceleration with values equivalent to $9.81\;m/s^{2}$ atan2 represent the arctangent function.

We used the three-axes of the raw accelerometer sensor, magnitude vectors, and rotational angle (pitch-roll) as inputs to the deep learning model. In addition, we evaluated the data both individually and when fused with each of the input features. We comprehensively conducted six experimental evaluations. These included the three-axis accelerometer, magnitude vectors, and pitch-roll angle. Then, we fused the magnitude vector, pitch and roll values to ensure thorough experimental assessment of the proposed methods. The data fusion were combined in the following processes: the three-axis accelerometer was column concatenated with magnitude vectors. The column concatenated data (three-axis accelerometer, magnitude vectors, and pitch-roll values), all computed from the combined pocket, and wrist acceleration sensors were used as input to the proposed deep learning methods. The six experimental evaluation are itemized below:

(1) Three-axis accelerometer data;
(2) Magnitude vector of the three-axis accelerometer data;
(3) Pitch-roll of the accelerometer data;
(4) Three-axis accelerometer data and computed magnitude vector;
(5) Three-axis accelerometer and computed pitch-roll;
(6) Three-axis accelerometer concatenated with magnitude vector, and pitch-roll angle.

### 3.4. Comparison with Conventional Machine Learning Methods

We compare the results obtained from the deep stacked autoencoder with the support vector machine classifier (SVM), Naïve Bayes classifier (NB), and linear discriminant analysis classifier (LDA). The support vector machine (SVM) is a powerful classification algorithm introduced in [59] for data classification. It uses hyperplane to separate training data using maximal margin. Support vector machine has been implemented for human activity recognition with various performance results, reported in literature [60,61].

On the other hand, the Naïve Bayes classifier is an efficient machine learning algorithm that utilize the Bayes theorem, with strong independent assumption for data classification and pattern recognition. Naïve Bayes is simple, fast, and produces high accuracy comparable to other classification implemented for human activity recognition [61]. While linear discriminant analysis classifier (LDA) is a linear classification algorithm that is highly optimized for multi-class classification problems. LDA is simple and works with high-dimensional data. The classification algorithm utilizes the assumption that sensor data have the same variance in order to estimate the mean and variance of the data for each class label. Therefore, the algorithm predicts the activity class label by estimating the probability that the new training data belong to each activity class label [62]. In their recent studies, [6] noted that linear discriminant analysis produce similar results with other classification algorithms, such as support vector machine, decision tree, and K nearest neighbors.

To evaluate the conventional machine learning, we extracted time domain features, such as mean, standard deviation, minimum, maximum, semi-quartile range, and median, as reported in [7]. We segmented the data using 2 s window size without overlap, and the extracted features were passed as training data to the support vector machine classifier, Naïve Bayes classifier, and linear discriminant analysis classifier. In addition, we implemented the protocol for pre-processing, feature extraction, and classification using MATLAB, 2017b statistical and machine learning toolbox. We utilized default parameter values for each of the classification algorithms discussed above.

## 4. Experimental Design

### 4.1. Dataset Description

The dataset used to evaluate the proposed deep learning-based complex human activity identification was collected in [7]. During dataset collection, 10 subjects attached Samsung Galaxy S2 smartphones on their wrists, and pockets, while performing a series of thirteen activities. These activities include jogging, walking, standing, sitting, biking, walking upstairs, and walking downstairs, performed for three min. Each subject also performed other activities, such as eating, typing, writing, drinking coffee, smoking, and giving talks for 3 to 5 min. The list of the performed activities, with range of activity duration, are shown in Table 2. The dataset contains a total of 1,170,000 instances of motion sensors, such as accelerometer, gyroscope, magnetometer, and linear acceleration, collected at 50 Hz sampling rate. During data collection, seven subjects out of a total of ten (10) subjects used in the experiments performed activities, such as eating, typing, writing, drinking coffee, and giving talks, while six subjects were smokers. In addition, all of the activities were performed indoors, except smoking and biking activities, which were carried outdoors. All of the subjects were males between the ages of 25 and 35. Activities, such as walking downstairs and upstairs, were performed at the university building, and the subjects were instructed to climb a series of five stairs, while activities, such as sitting and standing, were performed for 3 min without talking.

During typing and writing, seven subjects wrote a series of texts using laptop computers, and wrote on A4 paper. Shoaib, et al. [7] noted that only six subjects were smokers, and smoked one cigarette while sitting outside the building. Activities, such as smoking and drinking coffee, were carried to detect bad habits, physical inactivity, and lack of nutrition, which may adversely affect the overall well-being of the individual. In the present study, the accelerometer sensors collected using the wrist and pocket were utilized to evaluate the deep learning-based complex activity identification framework. The summary of the dataset description and pre-processing methods applied is shown in Table 2. The dataset present a unique means of evaluating human activity identification for enhanced smart healthcare due to the variety of activities performed. These include static, dynamic, and complex activity details.

### 4.2. Experimental Settings and Parameter Selection

The signal pre-processing, segmentation and deep stacked autoencoder methods were implemented with MATLAB, 2017b (https://in.mathworks.com/) using a system computer running on a Windows 10 operating system. The system was installed with Intel Core^TM^ I7-6700 CPU @ 3.400 GHz and random access memory (RAM) capacity of 16 GB.

In this study, the training window contains 100 samples after segmentation for the three-axis accelerometer data, magnitude vector, and pitch-roll values. We divided the pre-processed sensor data into two parts: 70% of the dataset was reserved for training while 30% for testing the developed model. Using this approach, the training sample contains 8190 instances while the testing set consists of 3510 instances for each axis of the acceleration sensor data, following a recent deep learning method for human activity recognition [8].

One of the major considerations in deep learning implementation is parameter selection. There is no theoretical method to determine each parameter during model implementation. Therefore, researchers conduct various experiments to assess the parameter combinations that achieve best performance within the context of the data [26,63]. The proposed deep stacked autoencoder has many parameters that require careful tuning to achieve improved performance results. In this paper, we optimize parameters, such as number of units in each hidden layer, training epoch, and sparsity terms using grid search methods. To select the best parameter, we used three-axis accelerometer data with three (3) hidden layers as a validation set. Table 3 itemizes the selected parameters, while the parameters are explained below.

**Figure 3:**
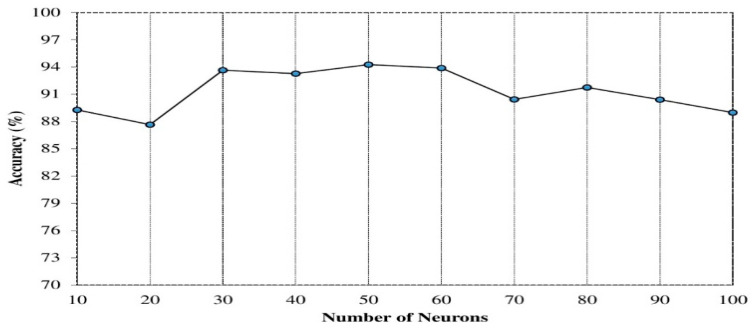
Impact of hidden neuron on the accuracy.

**Figure 4:**
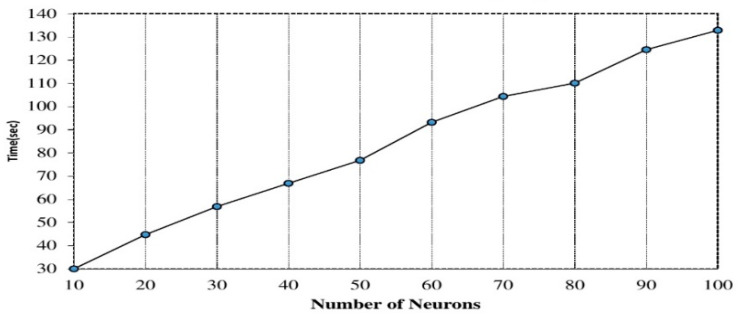
Computational time on increasing the hidden neurons.

The number of hidden neurons: specifically, we evaluated the number of hidden neurons between 10 and 100, and observed varied performance accuracies. Figure 3 shows the accuracies on utilizing different numbers of hidden neurons. The optimal accuracy was achieved for hidden neurons between 30 and 60. The need to vary the number of hidden neurons was informed by the fluctuating nature of the performance accuracies obtained at each iteration and recent implementation of deep learning for human activity identification [37]. However, we noticed that increasing the hidden neurons resulted in higher computational time, and may not improve the performance results [64] as shown in Figure 4. In our experiment, we set the first layer hidden neuron to 60, the second hidden layer to 40, and the third hidden layer to 30, respectively.

**Figure 5:**
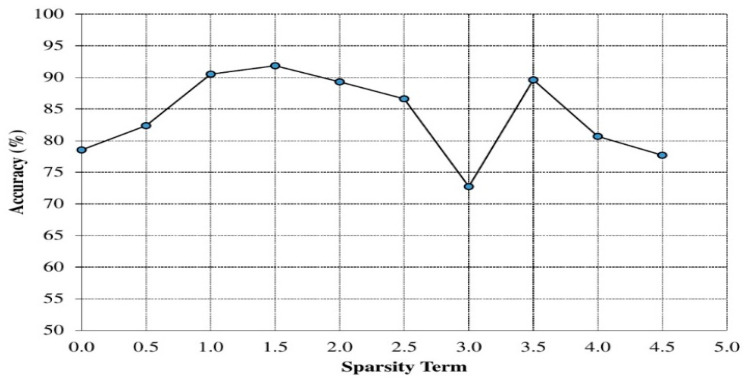
Influence of sparsity term on the performance results.

Sparsity terms: Figure 5 shows the dependency between sparsity of the sparsity autoencoder and accuracy. In this case, maximum accuracy was found when the sparsity term was set to 1.5; the value was used throughout the experiments.

**Figure 6:**
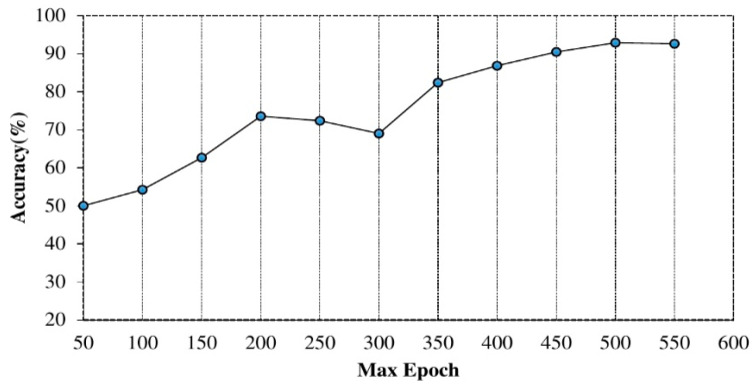
Max epoch Versus Accuracy.

**Figure 7:**
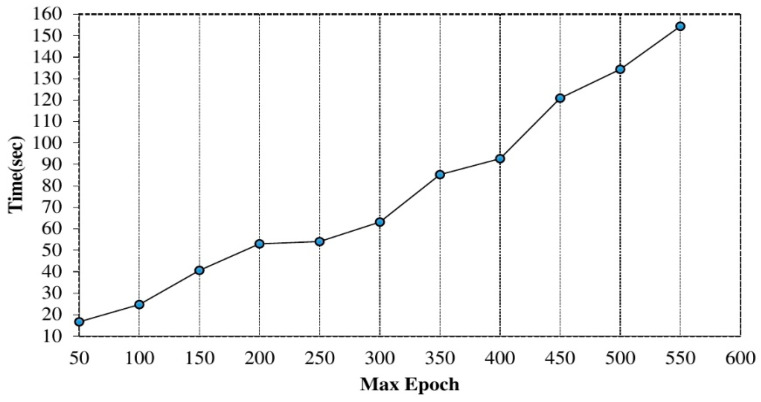
Computation time on increasing the max epoch.

The value of maximum epoch: furthermore, the significance of using different epoch on training the proposed model was evaluated, as shown in Figure 6. We started with fifty (50) epoch values and increased the values at various iterations. The max epoch values considered were 50 to 550 (interval of 50) and observed improved performance using higher epoch values. However, there is a correlation between higher max epoch and computation time as shown in Figure 7. The value of max epoch was set to 500 for each hidden layer.

*L*_2_ Regularization: the value of the loss cost function coefficient ($L_{2}$ regularization) is considered as an important parameter for training deep learning algorithms. To compensate for optimal convergence and good performance, we selected a learning rate of 0.001. We implemented other parameters of the proposed model using default values, using MATLAB, 2017b documentation on stacked autoencoder algorithms (https://in.mathworks.com/). To evaluate the optimal parameters for the proposed model, we use the data from 3-D acceleration (3-axes) as a validation set.

### 4.3. Evaluation Metrics

To evaluate the performances of the proposed deep learning-based complex human activity identification framework, we computed five (5) performance metrics. These include accuracy, recall, precision, f-measure, and error rate. For each activity class, the predicted values were measured with the ground truth label. We calculated the number of true-positive (TP), true-negative (TN), false-positive (FP), and false negative (FN) with the aid of confusion matrix of each prediction after testing. These performance metrics were chosen based on their wide applications for evaluating the performance of the human activity identification framework [29]. Here, N represents the total number of classes in the training sample.

Accuracy computes the rate of correctly classified activity classes out of the total number of activity instances. Accuracy is calculated using Equation (19).

(19) $$\frac{1}{N}\sum_{i=1}^{N}\frac{{\left(TP+TN\right)}_{i}}{{\left(TP+FP+TN+FN\right)}_{i}}$$

Recall represents the average number of the correctly predicted instance as positive instances. Recall rate is measured using Equation (20).

(20) $$\frac{1}{N}\sum_{i=1}^{N}\frac{{\left(TP\right)}_{i}}{{\left(TP+FN\right)}_{i}}$$

Specificity measures the average values of negative instances that are correctly classified from total number of activity instances. Specificity is computed, as shown in Equation (21).

(21) $$\frac{1}{N}\sum_{i=1}^{N}\frac{{\left(TN\right)}_{i}}{{\left(TP+FN\right)}_{i}}$$

## 5. Experimental Results and Discussion

In this section, various performance results obtained in each of the experimental settings are presented. The results are organized into two subsections: (1) the use of 3-D acceleration, magnitude (MAG), pitch and roll values, and (2) the fusion of 3-D acceleration, magnitude, pitch and roll values. In each evaluation, the performance results of the deep stacked autoencoder framework were compared with three classification algorithms (support vector machine, Naïve Bayes, and linear discriminant analysis), as explained in Section 3.4. The performance experiment is executed ten (10) times, and average results with standard deviation are reported.

### 5.1. Performance Results on 3-D Acceleration, Magnitude, Pitch and Roll Values

The results obtained on the performance results of each dataset used in our experiments are shown in Table 4. Three main performance metrics are used to analyze the performance of the proposed deep stacked autoencoder framework and baseline methods. From Table 4, when only the accelerometer sensor is used, the proposed deep stacked autoencoder framework achieved accuracy, recall, and specificity of 0.9292, 0.9290, and 0.9941, respectively. The use of pitch and roll values for the deep stacked autoencoder slightly outperforms 3-D acceleration and achieves accuracy and recall of 0.9297 and 0.9299, respectively. The performance results demonstrated with 3-D acceleration, pitch and roll, are significantly higher when compared with magnitude values. Due to lower dimensional values, magnitude values fail to achieve appreciable performance results. This is because the magnitude of acceleration sensors are fused with other sensor modalities to correct the effect of orientation invariance and displacements in smartphone-based human activity identification and authentication, and are rarely used in isolation for activity identification tasks [65]. Therefore, fusing magnitude with raw acceleration sensors would lead to improved recognition results. The performance of the proposed model is closely followed by Naïve Bayes with accuracy, recall, and specificity of 0.8420, 0.8423, and 0.9815, respectively. The lowest performance results are observed when the magnitude of the acceleration sensors is used for developing the deep stacked autoencoder algorithms. In this case, the use of Naïve Bayes classifier outperformed the proposed approach. The low performance of the proposed deep learning framework using the magnitude of the acceleration sensor is a result of less training data with few dimensions. To ensure optimal performance, deep learning requires high-dimensional data in order to learn robust feature vectors from the sensor data [66]. Thus, the use of training data with less number of dimensions, would make the stacked autoencoder learn features that are not efficient enough to identify activity details.

**Figure 8:**
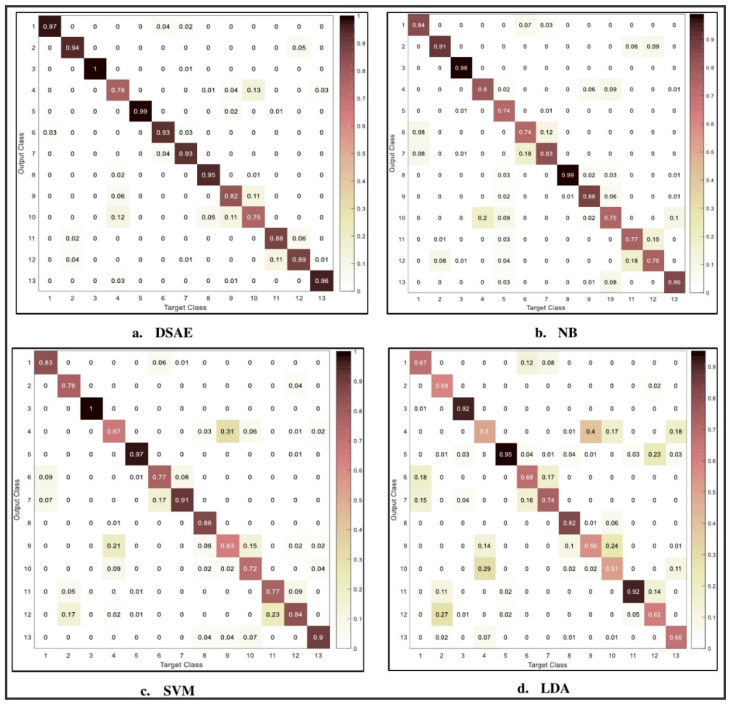
Confusion matrix showing the performance results of each method using the 3-D acceleration sensor (Note: DSAE (Deep stacked autoencoder), NB (Naïve Bayes), SVM (Support vector machine), LDA (Linear discriminant analysis). Activities: 1—walk, 2—stand, 3—jog, 4—sit, 5—bike, 6—upstairs, 7—downstairs, 8—type, 9—write, 10—coffee, 11—talk, 12—smoke, 13—eat.

Figure 8 shows the confusion matrix of 3-D acceleration using deep stacked autoencoder, Naïve Bayes, support vector machine, and linear discriminant analysis for complex human activity identification. The values in the confusion matrix are rounded to two decimal places for clarity and presentation. It can be seen from the confusion matrix that the use of the deep learning algorithm is effective for the identification of activity details, such as jogging, walking, biking, and eating. These activities are recognized with high accuracy when compared with other activities, such as sitting, taking coffee, and standing. Previous studies have shown that these activities are effectively recognized using orientation based sensor [7]. However, conventional machine learning algorithms are able to accurately recognize two to three activities compared to the proposed model. The activities accurately recognized include NB (jogging and typing), SVM (jogging and biking), and LDA (jogging, biking, and talking). These classification algorithms show a decrease in performance for other activity details accurately recognized by deep stacked autoencoder framework. In all, deep stacked autoencoder framework shows appreciable performances in both simple activities and complex activities that are difficult to identify with traditional machine learning methods [7]. Nevertheless, there is still room for improvement of these activities through the fusion of magnitude, pitch and roll values.

### 5.2. Performance Results on the Fusion of 3-D Acceleration, Magnitude, Pitch and Roll Values

In order to improve the results obtained with the implementation of 3-D acceleration, magnitude, pitch and roll, we evaluated the performances of the proposed deep learning model with the fusion of these values with the 3-D acceleration sensor. In each experimental evaluation, we fused magnitude with a 3-D acceleration sensor, pitch and roll with 3-D acceleration sensor and magnitude, pitch and roll with the 3-D acceleration sensor. The performance results obtained with these experimental settings are shown in Table 5. It can be observed that deep stacked autoencoder framework outperf ormed conventional classification algorithms such, as Naïve Bayes, support vector machine and linear discriminant analysis. The fusion of the acceleration sensor, magnitude, pitch and roll, demonstrated higher performance results. The use of orientation and rotation-based features provide enhanced performance results to the developed techniques for accurate detection of activity details. With the fusion, there are 2% to 51% improvements on accuracies when magnitude, pitch and roll values are fused with 3-D acceleration sensors. The fusion of orientation invariant and rotational angle based features show that the proposed fusion provides better and robust algorithms to enhance the complex human activity identification framework. The highest improvements on performance metrics are demonstrated against the use of Euclidean norm vectors. Specifically, there are observed improvement differences of 47.5%, 47.15%, and 3.95% on accuracy, recall, and specificity on 3-D acceleration with a fusion of Euclidean norm vector. Smartphone sensor performance is influenced by orientation sensitivity; incorporating magnitude and rotation angle (pitch and roll) would greatly reduce the effects and improve the robustness of the proposed framework, as can be seen in Table 5. These approaches have been collaborated by recent researchers [7,30] in human activity identification, using the conventional machine learning model. Similar performance results were demonstrated by conventional machine learning algorithms. For instance, there is a significant 5% increase in accuracy of the support vector machine with the fusion of Euclidian norm vector, pitch and roll values. However, the increase is not constant when compared to the deep stacked autoencoder framework. This can be observed in Naïve Bayes and linear discriminant analysis that provided a slight decrease in the same experimental scenario, as shown in Table 5.

**Figure 9:**
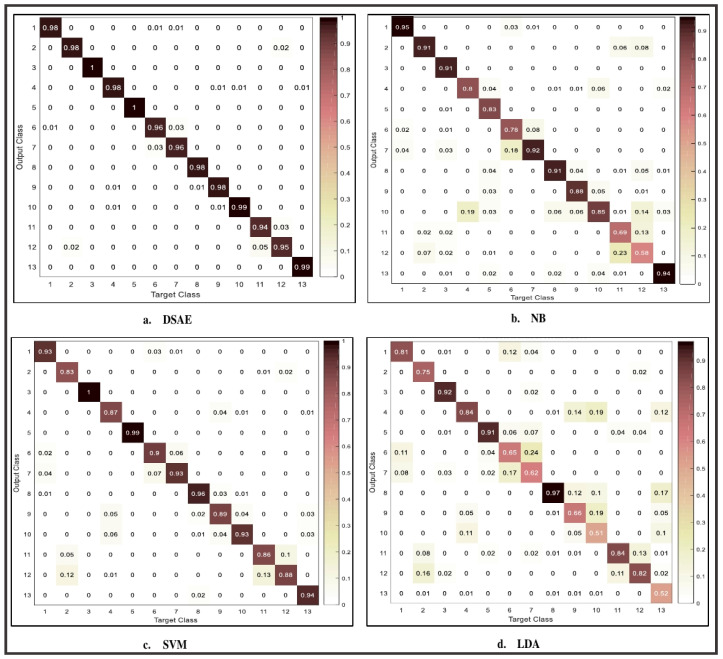
Confusion matrix for fusion of 3-D acceleration, magnitude, pitch and roll values. Note: DSAE (Deep stacked autoencoder), NB (Naïve Bayes), SVM (support vector machine), LDA (linear discriminant analysis). Activities: 1—walk, 2—stand, 3—jog, 4—sit, 5—bike, 6—upstairs, 7—downstairs, 8—type, 9—write, 10—coffee, 11—talk, 12—smoke, 13—eat.

Figure 9 shows the confusion matrix for the combination of 3-D acceleration, magnitude, pitch and roll values. The values in the confusion matrix are rounded to two decimal places for clarity and presentation. Due to limited space, we only depict the confusion matrix obtained by fusion of 3-D acceleration sensor, magnitude, pitch and roll values. From the confusion matrix, there are significant improvements in the performance of complex activity details when compared with using 3-D acceleration sensor discussed earlier. Activities, such as sitting, writing, taking coffee, and smoking, saw a significant increase in accuracy, between 6% and 24%. The performance revealed that the fusion of magnitude vector, pitch and roll, can provide enhancement to the framework and improve recognition of complex activity details. Moreover, activities that were recognized by the use of an acceleration sensor saw a significant boost in their performances. For instance, there is the complete identification of jogging, biking, taking coffee, and eating activities. These activities are important in maintaining a healthy lifestyle and reducing bad habits that may lead to health risks [7]. Furthermore, when compared with the conventional machine learning approach, there is less misrecognition between similar activity pairs. Using the Naïve Bayes classifier shows high misrecognition in activities, such as talking–smoking, sitting-taking coffee, and upstairs–downstairs. These activities are challenging to distinguish because they have similar patterns. Talking and smoking require the use of lip movements and hand gestures. Similarly, walking downstairs and upstairs are activities with a similar pattern and are difficult to identify using the accelerometer, conventional machine learning methods, and during activity labeling. Therefore, it requires the fusion of an accelerometer and orientation based sensor, such as a gyroscope and magnetometer, to achieve optimal performance. However, experimental evaluation with a deep stacked autoencoder shows better performances in the identification of these activities using 3-D acceleration, magnitude vectors, and pitch and roll values. The least performance among the conventional machine learning was demonstrated by linear discriminant analysis classifiers. This can be observed from the misclassification of most of the activity details shown in the confusion matrix (Figure 9). It is very challenging to identify activities, such as moving up and downstairs, writing, coffee, and eating. To recognize these activities may require the optimization of the classification algorithms or use of a higher number of feature. However, this may result in higher computation time and overfitting [29].

In a nutshell, with modern deep learning methods, robust feature vectors are hierarchically and automatically extracted from the sensor data, thereby, enhancing the recognition of complex activity details. The objectives of this paper are to develop an innovative deep learning method for complex human activity detection, and tackle rotation and orientation inconsistency in human activity detection using smartphone sensors. This is the first attempt at incorporating sensor rotation and displacement into deep learning-based complex human activity recognition. Extensive experimental evaluations were conducted to test the significance of the proposed model across the experimental setups; the deep stacked autoencoder consistently provided higher performance compared to Naïve Bayes, support vector machine, and linear discriminant analysis. The performance results obtained with the proposed fusion of 3-D acceleration, magnitude and rotation angle (pitch and roll) outstandingly outperformed conventional machine learning algorithms. The deep stacked autoencoder enables extraction of more discriminant features by transforming the smartphone sensor data into hidden features in order to reduce the error rate. Using the stacked autoencoder provided a mechanism to accurately distinguish complex activity details from high-dimensional data. In addition, the use of stacked invariant features provide ways to extract features that are invariant to orientation changes, distortion, displacement and placement positions [26]. Performance results obtained with this study demonstrate that fusion of rotation angle, magnitude with a raw acceleration sensor, can improve the complex human activity detection model. In particular, the implementation of the deep stacked autoencoder and data fusion method clearly shows significant improvements on challenging activities that are difficult to identify with acceleration signal alone [7]. These include complex activities, such as ascending stairs, descending stairs, drinking coffee, presenting talks, and smoking. With automatic feature representation using deep learning algorithms, the patterns of these activities were easily distinguished. The performance with the deep stacked method is consistent with previous studies on the use of deep learning for human activity recognition. These performance results are documented in a recent review on automatic feature representation for human activity recognition using deep learning algorithms [26]. Generally, the performance results suggest that the use of the proposed deep stacked autoencoder method is effective for identification of complex human activity and can guarantee better performance, especially with the current influx sensor data streams from ubiquitous smartphones and other wearable devices. In addition, deep stacked would reduce reliance on extensive feature selection to improve performance results in human activity identification.

### 5.3. Comparison with Deep Belief Networks

In order to demonstrate the effectiveness of the proposed deep stacked autoencoder for human activity identification, we compared it with another deep belief network (DBN). The Deep Belief Network (DBN) is a generative deep learning model for hierarchical extraction of discriminant features from data. The DBN method achieves hierarchical feature extraction by stacking multiple layers of the restricted Boltzmann machine and sigmoid belief network [67]. Typical deep belief network models have directed connection with the lower layers and undirected connection at the top of the layers. This allows DBN to extract observed distribution in training data using the stacked restricted Boltzmann machine [26]. In addition, DBN deploys layer-wise training alongside weight fine-tuning, using contrastive divergence to extract translational invariant features from sensor data. Deep belief networks have extensively been used in studies for human activity recognition and emotion identification [26,67]. In this study, the deep belief network was trained, each layer separately using contrastive divergence, as explained in [51], where the various parameters were intuitively considered during training. We set the number of hidden layers to two (2), and the number of epochs to 500. Other parameters we defined for the implemented deep belief networks include momentum = 0.5 and learning rate = 1.5.

Table 6 shows the results obtained using the deep belief network as compared with the proposed deep stacked autoencoder. From the table, it is obvious that the proposed deep stacked autoencoder outperformed deep belief networks. Using the individual data sample, deep belief network obtained lower performance results for 3-D acceleration, pitch and roll angle, except for magnitude vector, where the algorithm slightly outperformed the proposed deep stacked autoencoder, with performance difference between 1% to 13% for sensitivity, recall, and accuracy. Table 6 also revealed the results of deep belief networks (DBN) obtained for the fusion of various data samples. From the table, the deep stacked autoencoder (DSAE) algorithm significantly outperformed deep belief networks in all aspect of the experimental analysis. For the fusion of 3-D acceleration, magnitude, pitch and roll, we observed significant lower performance accuracy (91.57%), recall (91.66), and sensitivity (99.30%) for deep belief networks compared to the proposed deep stacked autoencoder. The results indicate that the proposed deep learning algorithm is suitable for extraction of discriminant features for enhanced identification of various details compared to conventional machine learning, and deep belief networks. The use of the deep autoencoder could save a significant amount of energy during feature extraction. The relevant features are automatically extracted from the raw sensor data [50].

**Figure 10:**
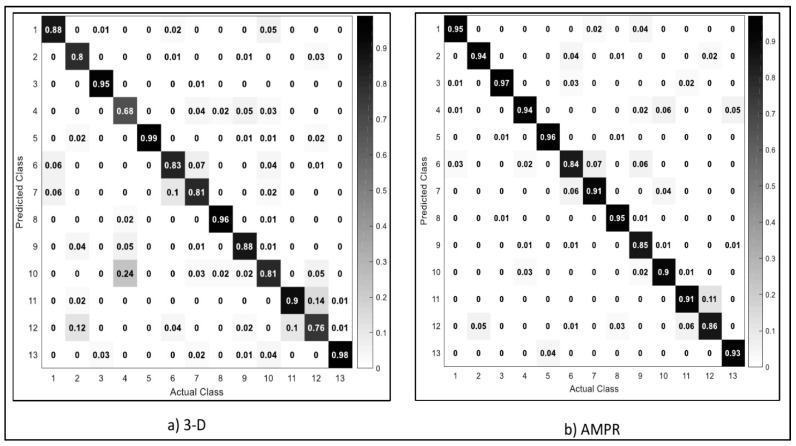
Confusion matrix for deep belief networks. Note: 3-D (3-D acceleration), AMPR (fusion of 3-D acceleration, Magnitude, pitch and roll). Activities: 1—walk, 2—stand, 3—jog, 4—sit, 5—bike, 6—upstairs, 7—downstairs, 8—type, 9—write, 10—coffee, 11—talk, 12—smoke, 13—eat.

We further analyze the performance results of individual activity as depicted in Figure 10. We observed that smoking activity (76%) achieved the lowest result for 3-D acceleration while similar results were obtained for climbing upstairs (84%) using the fusion 3-D acceleration, magnitude vectors, pitch and roll values. We observed a decrease in the recognition rate of activities accurately recognized by the deep stacked autoencoder. These include activities, such as biking, eating, walking, and walking upstairs, typing, and drinking coffee, for the fusion of all three data samples. However, deep belief networks clearly outperform conventional machine learning (NB, SVM, LDA) for recognition of individual activities, as shown in Figure 9 and Figure 10.

Another important comparison done for the proposed method is the computational complexity. The computational complexity of the proposed deep learning method depends on various factors, such as size of the dataset, number of hidden neuron, maximum epoch value, and computer used to run the experiments.

The system specification, experimental setting, and parameter selections of our experiments are discussed in Section 4.2. We computed the execution time of the experiments using the tic-toc function of MATLAB as shown in Table 7. The table shows the total time taken to preprocess, extract relevant features, and model activity details using each method. In addition, the experiments were ran ten (10) times to ensure statistical significance; the computational times shown are for fusion of all data samples. We observed that the proposed deep learning is of high computational complexity, and took a longer time to train when compared to conventional machine learning (NB, SVM, and LDA). However, the high computation complexity is worthwhile, compared to the significant increased error rate observed in conventional machine learning algorithms. Generally, deep learning models need more time to train due to the high number of hyper-parameter optimization required to achieve optimal results. The computational complexity of our proposed methods is less compared to deep belief networks.

## 6. Conclusions and Future Works

In this paper, we investigated the performance of the deep stacked autoencoder and orientation invariant feature to improve the performance of complex human activity identification framework. The proposed deep learning method is deployed to automatically extract robust and high representational features from motion sensor data. Furthermore, the paper proposes a fusion of magnitude vector, pitch and roll of the acceleration sensor, to correct the effects of orientation inconsistencies and position displacement inherent in smartphone-based complex human activity identification. Through extensive experiments using challenging accelerometer sensor data and complex activity details, the proposed approach achieved high accuracy on the identification of complex activities that are challenging to detect with traditional machine learning methods. In addition, the fusion of magnitude vector, pitch and roll, provided enhanced performance in terms of detection accuracy, recall, and sensitivity compared to state-of-the-art approaches for human complex/human activity identification. The proposed method clearly demonstrates the validity of the deep stacked autoencoder and orientation invariant feature augmentation to enhance complex human activity identification for smartphone implementation.

Although we have achieved high performance results using the deep stacked autoencoder, there are some limitations that may help improve the proposed activity identification framework. First, the fusion of the proposed approach with other sensor modalities, such as gyroscope, magnetometer, sound, pulse, and increasing the number of participants for data collection in order to provide comprehensive activity identification and health monitoring. The deep learning model performed better with a higher amount of data; therefore, collection of a huge amount of data will enable higher generalization of the results obtained. Second, deep learning algorithms require high parameter tuning to achieve optimal performance. The parameters utilized to develop the deep stacked autoencoder was selected by performance results on 3-D acceleration sensors. Deep learning algorithms perform differently in different experimental scenarios and training input samples, extensively testing the parameter selection on each training sample would improve the results. In most cases, training using such a procedure is time consuming, but would go a long way in selecting the best combination of deep stacked autoencoder parameters. In another scenario, there is still room to explore the proposed approach for on-board complex human activity using smartphones or smartwatches. Implementation of an efficient online-based deep stacked autoencoder would enable ubiquitous and seamless identification of activity details. Third, data augmentation exploit limited training data to enhance deep learning algorithms and avoid overfitting. Regardless, the proposed combination of 3-D acceleration with magnitude, pitch and roll, serve as data augmentation methods to reduce orientation inconsistency and displacement in smartphone devices. Other methods, such as arbitrary rotation, permutation of locations, scaling, and time warping, would greatly enhance complex human activity identification framework performances. Furthermore, we intend to utilize graphical processing units (GPUs) in the future to reduce training time of the deep neural networks.

## Figures and Tables

**Table 1:** Summary of various deep learning algorithms for human activity identification.

Deep Learning Methods	Descriptions	Strengths	Weaknesses
*Deep belief networks* [38,39,51]	Deep belief networks have direct connection to the lower layer of the network and hierarchically extract features from data.	Uses feedback mechanism to extract relevant features through unsupervised adaption.	High computation complexity due to high parameters initialization.
*Convolutional neural networks* [8,14,37,40,42]	Uses interconnected network structures to extract features that are invariant to distortion.	Widely utilized for human activity identification due to its ability to model time dependent data. It is invariant to changes in data distribution.	Requires large amount of training data to obtain discriminant features. In addition, it requires a high number of hyper-parameter optimization.
*Recurrent neural networks* [9,43,44]	Deep learning algorithm for modeling temporal changes in data.	Ability to extract temporal dependencies and complex changes in sequential data.	Difficult to train due to large parameter update and vanishing gradients.
*Deep autoencoder algorithms* [46,47,49,50]	Generative deep learning model that replicates copies of training data as input.	Reduces high-dimensional data to low dimensional feature vectors. This helps to reduce computational complexity.	Lack of scalability to high-dimensional data. It is difficult to train and optimize, especially for one layer autoencoder.

**Table 2:** Data description and pre-processing methods.

Activity Type	Activity List	Activity Durations
Static	Sitting	3 min
	Standing	3 min
Dynamic	Walking	3 min
	Jogging	3 min
	Biking	3 min
	Walking Upstairs	3 min
	Walking Downstairs	3 min
Complex Sequence	Eating	5 min
	Typing	5 min
	Writing	5 min
	Drinking Coffee	5 min
	Smoking	5 min
	Giving Talks	5 min

**Table 3:** Experimental Parameter Setting.

Parameters	Values
Hidden units	60-40-30
Sparsity terms (γ)	1.5
$L^{2}$ regularization (ψ)	0.001
Max epoch	500
Activation function (σ)	Sigmoid

**Table 4:** Performance results using each dataset.

	Accuracy			
	**DSAE**	**NB**	**SVM**	**LDA**
**3-D Acceleration**	0.9292 ± 0.0103	0.8420 ± 0.0048	0.8209 ± 0.0048	0.7194 ± 0.0147
**Magnitude**	0.4568 ± 0.0140	0.6050 ± 0.0087	0.4107 ± 0.0248	0.4160 ± 0.0080
**Pitch and Roll**	0.9297 ± 0.0053	0.7777 ± 0.0049	0.6202 ± 0.0082	0.4340 ± 0.0179
	**Recall**			
	**DSAE**	**NB**	**SVM**	**LDA**
**3-D Acceleration**	0.9290 ± 0.0107	0.8423 ± 0.0054	0.8202 ± 0.0049	0.7199 ± 0.0122
**Magnitude**	0.4601 ± 0.0107	0.6057 ± 0.0040	0.4107 ± 0.0239	0.4211 ± 0.0097
**Pitch and Roll**	0.9299 ± 0.0116	0.7778 ± 0.0093	0.6220 ± 0.0069	0.4356 ± 0.0170
	**Specificity**			
	**DSAE**	**NB**	**SVM**	**LDA**
**3-D Acceleration**	0.9941 ± 0.0009	0.9868 ± 0.0004	0.9851 ± 0.0004	0.9766 ± 0.0012
**Magnitude**	0.9548 ± 0.0012	0.9671 ± 0.0007	0.9509 ± 0.0020	0.9514 ± 0.0006
**Pitch and Roll**	0.9941 ± 0.0004	0.9815 ± 0.0003	96.84 ± 0.0007	0.9529 ± 0.0015

Note: DSAE (deep stacked autoencoder), NB (Naive Bayes), SVM (support vector machine), LDA (linear discriminant analysis), 3-D (three-axis dimensions).

**Table 5:** Performance results for fusion of different data samples.

Accuracy				
	**DSAE**	**NB**	**SVM**	**LDA**
3-D Acceleration and magnitude	0.9318 ± 0.0114	0.8419 ± 0.0041	0.8756 ± 0.0080	0.6983 ± 0.0173
3-D Acceleration and pitch and roll	0.9704 ± 0.0041	0.8265 ± 0.0034	0.8870 ± 0.0059	0.7071 ± 0.0158
3-D Acceleration, magnitude, pitch and roll	0.9713 ± 0.0041	0.8307 ± 0.0077	0.9084 ± 0.0049	0.7247 ± 0.0097
**Recall**				
	**DSAE**	**NB**	**SVM**	**LDA**
3-D Acceleration and magnitude	0.9316 ± 0.0116	0.8419 ± 0.0041	0.8763 ± 0.0069	0.7013 ± 0.0162
3-D Acceleration and pitch and roll	0.9703 ± 0.0041	0.8273 ± 0.0033	0.8875 ± 0.0062	0.7098 ± 0.0137
3-D Acceleration, magnitude, pitch and roll	0.9712 ± 0.0039	0.8305 ± 0.0073	0.9083 ± 0.0057	0.7265 ± 0.0092
**Specificity**				
	**DSAE**	**NB**	**SVM**	**LDA**
3-D Acceleration and magnitude	0.9943 ± 0.001	0.9868 ± 0.0003	0.9896 ± 0.0007	0.9741 ± 0.0014
3-D Acceleration and pitch and roll	0.9975 ± 0.0003	0.9856 ± 0.0003	0.9906 ± 0.0005	0.9756 ± 0.0013
3-D Acceleration, magnitude, pitch and roll	0.9976 ± 0.0003	0.9859 ± 0.0006	0.9924 ± 0.0005	0.9771 ± 0.0008

Note: DSAE (deep stacked autoencoder), NB (Naive Bayes), SVM (support vector machine), LDA (linear discriminant analysis).

**Table 6:** Comparison with deep belief networks (DBN).

Data	Methods	Accuracy	Recall	Sensitivity
**Performance Results on DBN using each data sample**				
*3-D acceleration*	*DSAE*	0.9292 ± 0.0103	0.9290 ± 0.0107	0.9941 ± 0.0009
	*DBN*	0.8612 ± 0.0090	0.8607 ± 0.0101	0.9884 ± 0.0002
*Magnitude*	*DSAE*	0.4568 ± 0.0140	0.4601 ± 0.0107	0.9548 ± 0.0012
	*DBN*	0.5821 ± 0.0054	0.5878 ± 0.0076	0.9652 ± 0.0008
*Pitch and roll*	*DSAE*	0.9297 ± 0.0053	0.9299 ± 0.0116	0.9941 ± 0.0004
	*DBN*	0.7630 ± 0.0132	0.7620 ± 0.0101	0.9803 ± 0.0006
**Performance Results on DBN using fusion of data samples**				
*3-D Acceleration and magnitude*	*DSAE*	0.9318 ± 0.0114	0.9316 ± 0.0116	0.9943 ± 0.0010
	*DBN*	0.8811 ± 0.0098	0.8824 ± 0.0086	0.9901 ± 0.0014
*3-D Acceleration and pitch and roll*	*DSAE*	0.9704 ± 0.0041	0.9703 ± 0.0041	0.9975 ± 0.0003
	*DBN*	0.8277 ± 0.0059	0.8261 ± 0.0054	0.9856 ± 0.0004
*3-D Acceleration, magnitude, pitch and roll*	*DSAE*	0.9713 ± 0.0041	0.9712 ± 0.0039	0.9976 ± 0.0003
	*DBN*	0.9157 ± 0.0077	0.9166 ± 0.0075	0.9930 ± 0.0005

**Note:** DSAE (Deep Stacked Autoencoder), DBN (Deep Belief Networks).

**Table 7:** Computational time of each method.

Methods	Execution Time (milliseconds)	Error Rate (%)
*Naïve Bayes (NB)*	22.7568	16.93
*Linear Discriminant analysis (LDA)*	18.4566	27.53
*Support vector machine (SVM)*	27.2124	9.16
*Deep Belief Networks (DBN)*	388.6811	8.43
*Proposed Method (DSAE)*	264. 7857	2.88

## Abbreviations

The following abbreviations were used in this paper.

Bi-LSTM	Bidirectional long short-term memory
CNN	Convolutional neural network
DSAE	Deep stacked autoencoder
FP	False positive
FN	False negative
GPU	Graphical processing unit
GPS	Global positioning system
IoT	Internet of Things
LDA	Linear discriminant analysis
LSTM	Long short-term memory
MAG	Magnitude vector
NB	Naïve Bayes
PCA	Principal component analysis
RAM	Random access memory
RNN	Recurrent neural network
SVM	Support vector machine
TN	True negative
TP	True positive
WHO	World Health Organization
